# Population and Public Health

**Published:** 1901-05-18

**Authors:** 


The Hospital

A Journal of

The Medical Sciences and Hospital Administration.

Vol. XXX.—No. 764.] Edited by Sir Henry Burdett, K.C.B., and Solomon C. Smith, M.D., M.R.C.P. [May 18, 1901.

### Population and Public Health.

Few public documents could be of a more entirely gratifying character than those which were issued last week by the Registrar-General, on the one hand in his capacity of Director of the Census, on the other in discharge of his more ordinary function as the chronicler of marriages, births, and deaths, among the population subject to his review. With regard to the Census, he tells us—speaking on the basis of figures which have not yet undergone final revision, and among which, therefore, there may lurk the possibility of trivial error—that the total number of persons in England and Wales, which increased by 3,028,086 between 1881 and 1891, has increased by 3,523,191 between 1891 and 1901, or almost precisely by half a million more than in the previous decade; while it would, we think, be impossible for any competent observer to doubt that the increase of population has been attended by a still greater increase of prosperity, or that the 32,000,000 of the present year are in the enjoyment of far more of the good things of life than fell to the share of the 25,000,000 of 20 years ago. The wage earner, especially, is not only better fed, better housed, better clothed, better educated, and better provided with the means of recreation than at any former period of our history, but is beginning, in a far greater degree than at any former period, to realise the magnitude of the national heritage into which he has fallen, and to recognise the duty of protecting and defending it. The increase of population is, indeed, not universal; insomuch that a decrease is recorded in thirteen out of the sixty-two administrative counties into which England and Wales are divided, while two are almost stationary. It is probable that nothing but local knowledge could fully explain these differences, more especially when we find, as in East and West Suffolk, or in the three divisions of Lincolnshire, an increase of population in one portion of a county and a decrease in another. It in no way follows, of course, that movements of population in the direction of decrease may not be on the whole beneficial to everybody concerned, migrations of labour which bring advantage alike to those who go and to those who remain behind; but the broad outlines of the Census which alone can yet be furnished to us do not afford the materials for any general conclusions upon such points as these. The question why East Suffolk has gained, in round numbers, 6,000 inhabitants, while West Suffolk has lost nearly 4,000, or why the Holland and Kesteven divisions of Lincolnshire have each lost about 1,400, while the Lindsey division has gained over 7,000, are no doubt capable of elucidation by those who are well acquainted with the conditions of local trade and industry; but, in the absence of these *data* they are matters which cannot be determined by any amount of ingenuity exerted in the direction of guessing. They will doubtless claim and receive, in due course, the careful attention of statisticians; and, in so far as losses of population may appear to arise from any conditions remediable by legislation, they will be likely ultimately to receive the consideration of Parliament. In the meanwhile they cannot be held to militate against the broad evidence of growing wealth and of increasing numbers.

When we turn to the other official document put forth from Somerset House—that is to say, to the Quarterly Return of Marriages, Births, and Deaths, we again find a series of figures which may be contemplated with complete satisfaction. The birth-rate has, indeed, been somewhat lower than in former years, having been only 29 per thousand of the population against a mean rate of 30·6 for the first quarters of the ten preceding years; but this does not by any means indicate that England and Wales have entered upon the downward course of decreasing fertility. The rustic epitaph over the grave of a baby—

> "If so soon that I was done for,
> I wonder what I was begun for?"

was the composition of a true philosopher, and the nation gains nothing by a fertility which is counterbalanced by premature decease. The future of a people does not so much depend upon the number of children born to them as upon the number reared to healthy and vigorous manhood or womanhood, and in this respect the story told by the Registrar-General is altogether encouraging. The mortality of infants in the past quarter, measured by the proportion of deaths under one year of age to registered births was 137 per thousand, the average of the ten preceding first quarters having been 147. The difference means that no less than 2,320 children who in recent years would have died have this year remained alive, and it is manifest that a change in this direction, which we have every reason to expect will be uniformly progressive, will at no distant period afford ample compensation for some small diminution in the birth-rate. Infantile mortality, as sanitary reformers have constantly had occasion to point out, affords the most delicate possible test of the condition of public health among the community in which it occurs. On the present occasion, however, we may not only congratulate ourselves upon the lowering of the infantile death rate, but also, and in a corresponding degree, upon the diminution of mortality at all other ages as well. The average total death-rate of 20·7 per 1,000 for the preceding 10 quarters, has this year fallen to 18·3; the rate for persons between one year and 60 to 9·2 from 10·5; and the rate for persons over 60 from 92·3 to 82·5. We fear it is impossible to expect that the practice of dying will ever become entirely obsolete; but everything tends to the hope that it will, more and more, be deferred to that period of life at which it comes as the natural sequel to work accomplished and duty done.

